# Drug Repositioning for Gynecologic Tumors: A New Therapeutic Strategy for Cancer

**DOI:** 10.1155/2015/341362

**Published:** 2015-02-03

**Authors:** Kouji Banno, Miho Iida, Megumi Yanokura, Haruko Irie, Kenta Masuda, Yusuke Kobayashi, Eiichiro Tominaga, Daisuke Aoki

**Affiliations:** Department of Obstetrics and Gynecology, School of Medicine, Keio University, Tokyo 160-0016, Japan

## Abstract

The goals of drug repositioning are to find a new pharmacological effect of a drug for which human safety and pharmacokinetics are established and to expand the therapeutic range of the drug to another disease. Such drug discovery can be performed at low cost and in the short term based on the results of previous clinical trials. New drugs for gynecologic tumors may be found by drug repositioning. For example, PPAR ligands may be effective against ovarian cancer, since PPAR activation eliminates COX-2 expression, arrests the cell cycle, and induces apoptosis. Metformin, an antidiabetic drug, is effective for endometrial cancer through inhibition of the PI3K-Akt-mTOR pathway by activating LKB1-AMPK and reduction of insulin and insulin-like growth factor-1 due to AMPK activation. COX-2 inhibitors for cervical cancer may also be examples of drug repositioning. PGE2 is induced in the arachidonate cascade by COX-2. PGE2 maintains high expression of COX-2 and induces angiogenic factors including VEGF and bFGF, causing carcinogenesis. COX-2 inhibitors suppress these actions and inhibit carcinogenesis. Combination therapy using drugs found by drug repositioning and current anticancer drugs may increase efficacy and reduce adverse drug reactions. Thus, drug repositioning may become a key approach for gynecologic cancer in drug discovery.

## 1. Introduction

The goals of drug repositioning are to find a new pharmacological effect for a drug for which human safety and pharmacokinetics are established and to expand the therapeutic range of the drug to another disease. The concept of drug repositioning is well known in Europe and the United States, but uncommon in Asia. Drugs for which indications have been expanded by drug repositioning include thalidomide, aspirin, metformin, and digoxin. Thalidomide is a sedative hypnotic agent that has serious teratogenic effects in administration to pregnant women [1]. However, thalidomide is effective against multiple myeloma and leprosy and is now used for these diseases [2–4]. Aspirin, a nonsteroidal anti-inflammatory drug (NSAID) with antiplatelet effects, has also been shown to be effective against colorectal cancer [5]; metformin, a drug for type 2 diabetes, is effective against many cancers [6]; and digoxin, a cardiac glycoside, has efficacy against prostate cancer [7].

In this paper, we discuss potential drug repositioning as a new therapeutic strategy for gynecologic tumors, including peroxisome proliferator-activated receptor (PPAR) ligands and ritonavir for treatment of ovarian cancer, metformin for treatment of endometrial cancer, and cyclooxygenase-2 (COX-2) inhibitors for cervical cancer.

## 2. Drug Repositioning as Drug Development

In the 1990s, the drug discovery process was improved by developments in genome-based drug discovery, high-throughput screening, and combinatorial chemistry, with the anticipation that many new drugs would be developed in the 21st century. However, the number of new drugs launched on the market has decreased year after year, due mainly to unexpected adverse reactions in clinical trials and poor human pharmacokinetics. Since drugs with safety and good pharmacokinetics have already been developed, drug repositioning may be useful as a new approach to drug discovery [8–11].

The concept of drug repositioning is based on the idea that low molecular weight compounds are unlikely to act on only one target among the many proteins in cells [12]. Drug repositioning is also facilitated by elucidation of molecular mechanisms involved in disease onset and progression, that is, common pathologies of different diseases that were considered to be unrelated to each other. Drug repositioning also takes advantages of new approaches such as DNA chips to facilitate a comprehensive investigation of the molecular pharmacology of existing drugs, with the goal of expanding the range of indications based on newly discovered pharmacological effects [12].

Drug repositioning has attracted attention due to significant advantages over traditional drug development. It targets not only drugs that are on the current market but also those that have been withdrawn because of adverse effects or those that have passed the safety issues in clinical trials but efficacy was insufficient and resulted in development failure. The available clinical trial data of suspended drugs reduces the time and costs of drug development. Since the human safety and pharmacokinetics of these drugs have already been evaluated, unexpected adverse reactions and abnormal pharmacokinetics are unlikely to occur in clinical trials. The reason behind the slow progress in new drug development is partly due to potential drugs showing efficacy and safety in animal studies, but not in humans. However, in drug repositioning, human safety is already established, and therefore candidate drugs from animal studies can be safely provisionally administered to humans to examine efficacy, which allows full-fledged development if efficacy is shown.

In Japan, drug repositioning was introduced around 2012. Drug repositioning in Europe and the United States is better established and this approach produced six drugs approved by the U.S. Food and Drug Administration (FDA) in 2009, compared to only one drug in 2001. New indications introduced by drug repositioning include thalidomide, a sedative hypnotic agent, for multiple myeloma and leprosy [2–4]; a COX-2 inhibitor for pancreatic cancer and colorectal cancer [13, 14]; aspirin, an NSAID, for colorectal cancer [5]; and metformin, an antidiabetic drug, for endometrial cancer [6]. In this paper, we examine potential drug repositioning of PPAR ligands and ritonavir for ovarian cancer, metformin for endometrial cancer, and a COX-2 inhibitor for cervical cancer.

## 3. PPARs and Ovarian Cancer

Peroxisome proliferator-activated receptors (PPARs) are nuclear receptor proteins that were discovered in 1990 [15]. Three subtypes of PPARs, *α*, *δ*, and *γ*, have been identified, which are expressed in different tissues [16]. PPAR*α* is expressed mainly in skeletal muscle, liver, kidney, and heart and plays an important role in fatty acid metabolism. PPAR*δ* is distributed in many tissues and regulates intracranial lipid metabolism, high-density lipoprotein (HDL) metabolism, adipogenesis, and preadipocyte differentiation. PPAR*γ* consists of three subtypes: PPAR*γ*1 is expressed in many tissues including heart, muscle, colon, kidney, spleen, and pancreas; PPAR*γ*2 is found in adipose tissues; and PPAR*γ*3 is found in macrophages, colon, and white adipose tissues. PPAR*γ* is involved in cell differentiation, adipose depots, and modification of insulin action. PPARs are activated by specific ligands and bind to regulatory regions of target genes to regulate gene expression. PPARs are targets in treatment of diabetes, dyslipidemia, and arteriosclerosis due to this gene regulation mechanism. PPARs are also expressed in various cancer cells and are implicated in oncogenesis.

PPARs play a direct role in ovarian physiology through regulation of expression and activation of proteases influencing tissue reconstruction and angiogenesis in follicular development, ovulation, and luteinization [17–20]. Komar et al. showed expression and localization of PPARs in normal rat ovarian tissues using* in situ* hybridization and specific roles of PPARs during follicular development [21], with localization of PPAR*γ* in granulosa cells and PPAR*α* and PPAR*δ* in the capsule and stroma. PPAR*γ* was shown to be regulated by luteinizing hormone, which is maintained at a high level during follicular development and decreases with ovulation. Furthermore, progesterone and estrogen were markedly increased by administration of ligands for PPAR*γ*. However, PPAR*α* and PPAR*δ* were expressed at high levels regardless of the estrous cycle, indicating that PPAR*α* and PPAR*δ* are involved in fundamental aspects of ovarian function, the details of which are unknown. Thus, ligands specific to PPARs have certain effects on ovarian function, but the mechanisms are unclear.

In 2004, Nicol et al. found that PPAR*γ* inhibited ovarian carcinogenesis induced by the carcinogen dimethyl benzanthracene (DMBA) in mice [22]. Following administration of DMBA, the incidences of cancer and metastasis in PPAR*γ* heterogenous knockout mice were ≥3-fold and 4.6-fold those in PPAR*γ* wild mice, respectively. This suggests that PPAR*γ* reduces ovarian carcinogenesis. Also in 2004, Sakamoto et al. showed that overexpression of COX-2 and underexpression of PPAR*γ* in ovarian epithelial cells were strongly implicated in ovarian carcinogenesis and that PPAR*γ* activation in ovarian cancer cells inhibited COX-2 expression via the nuclear factor-kappa B (NF*κ*B) pathway [23]. COX-2 is involved in carcinogenesis of colorectal and breast cancer, and activation of PPAR*γ* by specific ligands eliminates COX-2 expression induced by tumor necrosis factor (TNF)-*α* [23]. Therefore, PPAR*γ* expression is inversely correlated with COX-2 expression.

**Figure 1:**
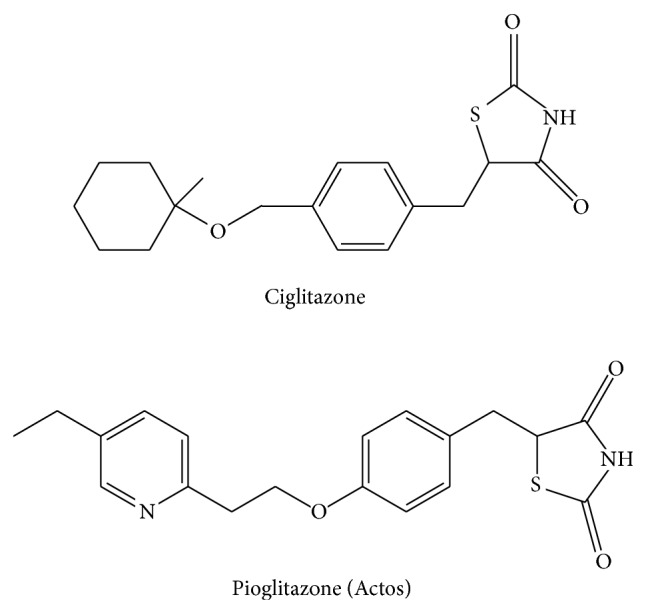
Structures of ciglitazone and pioglitazone [25].

These results suggest that ligands activating PPAR*γ* may inhibit ovarian carcinogenesis and serve as a therapeutic strategy for ovarian cancer. Several studies have found relationships between PPAR*γ* ligands and ovarian cancer. These ligands include ciglitazone, which inhibits cell growth by causing cell cycle arrest and apoptosis in ovarian cancer cells [24, 25] (Figure 1), and DIM-C-pPhtBu, which arrests the cell cycle by inducing PPAR*γ*-dependent p21. DIM-C-pPhtBu also reduces the activity of PPAR*γ*-independent cyclin D1 and induces apoptosis, which consequently inhibits cell growth [26]. In an* in vivo* study in mice with subcutaneous ovarian tumors and cancerous peritonitis, Xin et al. found that direct injection of ciglitazone significantly prolonged the survival of mice with cancerous peritonitis [27]. PPAR*γ* in subcutaneous tumors of mice treated with ciglitazone significantly increased in comparison with that before administration. Apoptosis was induced and angiogenesis was inhibited in tumors treated with ciglitazone. Ciglitazone did not change COX-2 levels in tumors, but microsomal prostaglandin E synthase (mPGES) markedly decreased. Thus, ciglitazone reduced prostaglandin E2 (PGE2) in a COX-2-independent manner, induced apoptosis, reduced angiogenesis, and inhibited tumor progression.

Pioglitazone, another PPAR*γ* ligand, has similar inhibitory effects on tumor progression [28]. However, a clinical trial showed that the incidence of bladder cancer with pioglitazone was higher than that with placebo [29]. The relationship of bladder cancer and pioglitazone has been examined in the Kaiser Permanente Northern California (KPNC) study and in various meta-analyses, with different results. In 2014, the European Medicines Agency (EMA) recommended that pioglitazone should not be given to patients with bladder cancer or a history of bladder cancer. The FDA also instructed avoidance of pioglitazone for patients during treatment of bladder cancer because pioglitazone for one year or more increased the risk of onset of bladder cancer. The mechanism of action of pioglitazone is unclear and the risks associated with bladder cancer require further evaluation.

Fibrates are lipid-lowering drugs that activate PPAR*α* and promote lipid metabolism, which increases HDL cholesterol and has a preventive effect on arteriosclerosis. An* in vitro* study in 2006 showed that PPAR*α* ligands inhibit cancer cell growth [30] and several subsequent studies have shown that PPAR*α* activation inhibits tumor growth [31–33]. These results confirm a relationship of PPAR*α* with tumor growth. Grau et al. found that ligand-based PPAR*α* activation in colorectal cancer cells inhibited transcriptional induction of COX-2 and vascular endothelial growth factor (VEGF) [30], with the mechanism thought to involve inhibition of induction of activator protein-1- (AP-1-) dependent genes, which are involved in tumor progression. AP-1 expression is regulated by the oncogene c-Jun. In tumor growth inhibition, activated PPAR*α* may bind directly to consensus DNA sequences, which inhibits c-Jun transcriptional activity and attenuates AP-1 expression. A subsequent study using PPAR*α* ligands showed downregulated AP-1 expression in ovarian cancer cells and inhibition of development of ovarian cancer, providing support for this mechanism [28].

Inhibition of solid tumor growth* in vivo* by PPAR*α* activation was first shown in 2007 [31]. Cancer-bearing mice and mice with cancerous peritonitis were produced using two types of human ovarian cancer cells and treated with clofibric acid, a PPAR*α* ligand. Clofibric acid reduced the tumor size and prolonged survival equally or more effectively compared to cisplatin, which is used for chemotherapy of ovarian cancer. The main mechanism of action may involve carbonyl reductase, which is induced in tumors by clofibric acid. Carbonyl reductase is an enzyme that metabolizes carbonyl compounds with use of nicotinamide adenine dinucleotide phosphate (NADPH) [34]. Clofibric acid also converts PGE2 to prostaglandin F2*α* (PGF2*α*) [35], and PGE2 induces inflammation, promotes angiogenesis, and inhibits apoptosis and consequently is implicated in tumor growth [36, 37]. Clofibric acid also directly decreases the level of mPGES, a PGE2 synthase. Thus clofibric acid may reduce PGE2 activity in ovarian cancer by increasing carbonyl reductase and decreasing mPGES, with resultant inhibition of angiogenesis and induction of apoptosis. Inflammation occurs around tumors and inflammatory cells release angiogenic factors and cytokines, which serve as nutrients for tumor cells [32, 33]. PGE2 induces inflammation, and ligand-induced PPAR*α* activation inhibits this inflammation around tumor cells.

Few studies have evaluated the relationship of PPAR*δ* with malignant tumors. PPAR*δ* activation inhibits cell growth in breast cancer, lung cancer, and melanoma cells* in vivo* [38, 39], but the mechanisms of action are inconsistent. Daikoku et al. neutralized PPAR*δ* in ovarian cancer cells overexpressing PPAR*δ in vivo* and found tumor growth inhibitory effects [40]. Aspirin, a COX-1 selective inhibitor, inhibits growth of ovarian cancer and attenuates PPAR*δ* function, implicating PPAR*δ* inactivation in growth inhibition of ovarian cancer [40]. However, another study showed that aspirin did not inhibit ovarian cancer growth [41] and further studies are required to examine the role of PPAR*δ* in malignant tumors.

## 4. Ritonavir and Ovarian Cancer

Ovarian cancer is a gynecological malignant tumor with a high mortality because it is difficult to detect at an early stage. More than half of patients with ovarian cancer who undergo chemotherapy with a first line platinum agent do not achieve clinical remission completely [42].

Treatment of HIV infection has the goal of prevention or control of acquired immune deficiency syndrome (AIDS). In antiretroviral therapy for inhibition of HIV growth, anti-HIV drugs targeting all viral life cycle stages are used. There are five types of currently approved anti-HIV drugs: nucleoside analogue reverse transcriptase inhibitors (NRTI), nonnucleoside analogue reverse transcriptase inhibitors (NNRTI), protease inhibitors (PI), integrase inhibitors (INI), and invasion inhibitors. Monotherapy with these drugs produces resistant viruses, resulting in failure of treatment; therefore, it is common to use three or four drugs, such as in highly active antiretroviral therapy (HAART). Typical HAART is a combination of NRTIs and PIs. HAART reduces onset of AIDS and opportunistic infection and risks for hospitalization and death [43]. Interestingly, the incidence of tumor lesions also decreases in patients treated with HAART [44–46]. Clifford et al. conducted a cohort study of HIV and found that the standardized incidence ratio of Kaposi's sarcoma was significantly lower in patients treated with HAART (25.3, 95% CI: 10.8–50.1) compared to those treated without HAART (239, 95% CI: 211–270) [47]. Other studies have shown similar results for the antitumor effect of HAART [45, 48, 49].

Ritonavir, a PI used for HAART, induces apoptosis of lymphoblastic tumor cells including lymphoma, myeloid leukemia, fibrosarcoma, and mastocytoma, as well as Kaposi's sarcoma [50, 51]. Ritonavir has an antitumor effect on MDH-2774 and SKOV-3 ovarian cancer cells through dose-dependent inhibition of the cell cycle and induction of apoptosis. After administration of ritonavir at a dose of 20 *μ*M for 3 days, cell death was induced in more than 60% of MDH-2774 cells and 55% of SKOV-3 cells. Ritonavir arrests the G1 phase of the cell cycle in ovarian cancer cells by depleting retinoblastoma (RB) phosphorylation, G1 cyclin, and cyclin dependent kinase. The phosphatidylinositol-3 kinase- (PI3K-) protein kinase- B(Akt-) mammalian target of rapamycin (mTOR) signaling pathway is required for cell growth and plays an important role in progression and metastasis of various cancers [52, 53]. Ritonavir dose-dependently decreases the amount of phosphorylated Akt, which inhibits the PI3K-Akt pathway and has an antitumor effect. Akt inhibition by siRNA increases the apoptotic effect synergistically with ritonavir. These results suggest that use of ritonavir in combination with an Akt inhibitor may enhance the antitumor effect. The efficacy of this approach compared to conventional chemotherapy requires further evaluation.

## 5. Metformin and Endometrial Cancer

Patients with type 2 diabetes have risks of cancer including hepatic, endometrial, pancreatic, colon, bladder, and breast cancer [54, 55]. Most cases of endometrial cancer are adenocarcinoma and have low radiosensitivity; thus, the first treatment option is surgical therapy since there is little effective chemotherapy. Progestin therapy provides an option for fertility preservation and has a relatively high response rate and few adverse reactions; however, recurrence is high and the therapeutic effect is limited. The National Comprehensive Cancer Network (NCCN) Clinical Practice Guidelines in Oncology [56] recommend indications of progestin therapy for young patients with atypical endometrial hyperplasia (AEH) or Grade 1 endometrioid adenocarcinoma who want fertility preservation and for patients ineligible for surgery [57–59]. The response rate of hormone therapy is high if patients meet these criteria. If an appropriate response is not obtained, many patients who undergo hysterectomy as a change of therapeutic strategy achieve complete remission (CR). Conserving therapy uses three types of progesterone drugs: hydroxyprogesterone caproate (HPC), medroxyprogesterone acetate (MPA), and megestrol acetate (MA). A levonorgestrel intrauterine device (LNG-IUS) has also recently been used.

In a meta-analysis published in 2012, Gallos et al. showed that the response to conservative therapy using progesterone was 76% in endometrial cancer and 86% in AEH, which are relatively high [60]. However, the recurrence rates after remission were 41% in endometrial cancer and 26% in AEH, which are also high [60]. The median disease-free survival until recurrence was 24 months [61] and most cases recurred one to three years after treatment. Consequently, only 32% of patients with endometrial cancer had long-term remission [62]. The high recurrence rate after conservative therapy using progesterone indicates that a new therapeutic strategy is required. These may include LNG-IUS+GnRH therapy [63] and photodynamic therapy, a procedure for irradiating red light at 630 nm in the uterine cavity after intravenous injection of photosensitizers [64]. However, outcomes are currently insufficient and these approaches are not an alternative to progestin therapy.

Metformin, a drug for type 2 diabetes, is of interest in drug repositioning for endometrial cancer. In an epidemiological study, Evans et al. found that the incidence of endometrial cancer in diabetic patients treated with metformin was lower than that in patients who were not given metformin [65]. Metformin is an oral drug of the biguanide class that is commonly used in patients with diabetes, in addition to exercise and diet therapy. Metformin inhibits gluconeogenesis, enhances insulin sensitivity by inducing glucose transporters, normalizes blood glucose by increasing glucose uptake in skeletal muscles, and decreases serum insulin. Sulfonylureas stimulate insulin secretion and decrease blood glucose, while metformin decreases insulin secretion and blood glucose.

As described above, endometrial cancer is strongly related to obesity and many patients with endometrial cancer have hyperalimentation and high blood insulin [66]. The function of phosphatase and tensin homolog (PTEN) is frequently disturbed in endometrial cancer cells. Consequently, the PI3K-Akt-mTOR signaling pathway is activated in many patients with endometrial cancer. Several mechanisms of action for the antitumor effect of metformin have been proposed: activation of LKB1-AMP-activated protein kinase (LKB1-AMPK), which inhibits the mTOR pathway, with resultant induction of progesterone receptor (PR) expression and recovery of progesterone sensitivity [67, 68]; direct reduction of insulin and insulin-like growth factor-1 (IGF-1) due to AMPK activation; angiogenetic inhibition by reducing VEGF; cell cycle arrest by inhibiting expression of mitogen-activated protein kinase (MAPK) and cyclin D1; and inhibition of expression of epithelial growth factor receptor 2 (HER2) and HER2 protein kinase and inhibition of downstream signaling [69].

In 2014, Mitsuhashi et al. conducted a study in 31 patients with endometrial cancer who were scheduled to undergo surgery and were given metformin prior to surgery. Growth of tumor cells in the endometrium was compared histologically before administration to after hysterectomy [70]. There was a significant decrease of 44.2% (95% CI: 35.4–53.0; *P* < 0.01) in the positive rate for Ki-67, a cell cycle protein that is correlated with the tumor grade. AMPK increased by 113.2% (95% CI: 13.6–212.8; *P* = 0.03) and AMPK activation inhibits the mTOR pathway, resulting in decreased activity of downstream substrate ribosomal protein S6 (rpS6). Inhibition of MAPK and p27 expression increased by 59.0% (95% CI: 12.5–105.5; *P* = 0.02). These results show that metformin inhibits malignant growth of endometrial cancer* in vivo*.

Mitsuhashi et al. evaluated the effect of a combination of metformin with MPA, which is used in conventional progestin therapy [70]. Metformin at a dose of 1500–2250 mg/day was administered to patients with AEH or juvenile endometrial cancer (FIGO stage IA, G1 endometrioid adenocarcinoma) who wanted fertility preservation, simultaneously with MPA at a dose of 400 mg/day. MPA was administered for 6 to 9 months and 91% of patients had CR. Metformin was administered continuously after completion of MPA administration. An interim analysis conducted after a median observation period of 26 months showed a recurrence rate of only 5%. Given that the recurrence rate after MPA therapy is 40–50%, metformin appears to be effective for prevention of cancer recurrence. Diarrhea and nausea (Grade 2 or higher) were observed in 13% of patients during the study; however, these symptoms were relieved by decreasing the metformin dose and did not require suspension of metformin administration. Metabolic acidosis is also a known adverse reaction of metformin when used as an antidiabetic drug. However, metformin is considered to be relatively safe for young women without renal dysfunction, and no serious adverse reactions were observed. Body weight gain is also an adverse reaction of MPA, but this was suppressed by metformin and body weight was reduced after completion of MPA administration. Administration of metformin also improved insulin resistance and reduced IGF-1. Based on these results, MPA+metformin therapy resolves several problems of MPA monotherapy, including a high recurrence rate, and has favorable effects on inhibition of body weight gain, an adverse reaction of MPA, and improved insulin resistance. Therefore, metformin may be a new therapy for younger patients with endometrial cancer, although further clinical trials are required.

## 6. COX-2 and Cervical Cancer

**Figure 2:**
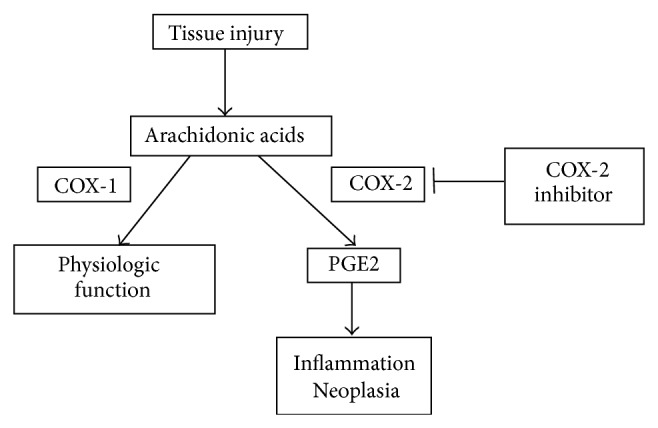
Cyclooxygenase pathways.

COX is a kinase enzyme that converts arachidonic acid released from phospholipids of cell membranes by phospholipase A2 (PLA2) to prostaglandins (PGs) and other eicosanoids (Figure 2). COX-2 is an inducible enzyme that is transiently produced in the nucleus by stimulation of interleukin 1 (IL-1), the primary inflammatory cytokine, and TNF-*α* when inflammatory cells are activated. COX-2 is expressed in inflammatory cells including macrophages, neutrophils, fibroblasts, and synoviocytes, and at least 1 to 2 hours is required for onset of the enzyme activity. Two transcription factors, AP-1 and NF-*κ*B, play major roles in inducing COX-2 expression following stimulation by growth factors, cytokines, hormones, and endotoxins. COX-2 is involved in inflammation, vasodilation, bone resorption, cancer growth, angiogenesis, gastric ulcer repair, and granulation [71, 72]. An increase in PGE2, the main product of COX-2, increases vascular permeability and the vascular effusion response in early inflammation. COX-2 is expressed at extremely low levels in most tissues under normal physiological conditions and is induced by response of inflammatory stimulation. Thus, COX-2 is mainly expressed in inflammatory and immunocompetent cells, and the amount of PG production by COX-2 is greater than that of COX-1. Therefore, COX-2 is involved in pathogenic malignancies and possible roles of COX-2 in carcinogenesis are of interest.

Tumor and normal tissues have no significant difference in COX-1 expression, but COX-2 has significantly higher expression in tumor tissues, including digestive cancers such as colon, gastric, esophageal, and pancreatic cancer, and in lung, breast, bladder, cervical, and head and neck cancer and brain tumors [73]. High COX-2 expression is found in early to advanced cancer and upregulation is particularly strong in patients with metastasis and a poor prognosis [74]. In colorectal tumor, COX-2 overexpression occurs in 40–50% of cases of adenomatous polyposis and 80–90% of colorectal cancers [75]. COX-2 is highly expressed in interstitial fibroblasts and invasive inflammatory cells in adenomatous polyposis and in tumor epithelial cells and stromal cells in colorectal cancer [76]. COX-2 expression is also increased in lung cancer and particularly in lung adenocarcinoma. Rarely expressed in normal alveolar epithelial cells, COX-2 is overexpressed in 30% of cases of atypical adenomatous hyperplasia, a precancerous lesion, and in 70% of lung adenocarcinomas [77]. Among patients with stage I lung cancer who underwent radical surgery, the five-year survival rate was significantly higher in COX-2-negative cases (88%) compared to cases with COX-2 overexpression (66%) [78].

The incidence of COX-2 expression in breast cancer ranges from 5% to 90%, with high expression in patients with a poor prognosis [79], highly malignant breast cancer [80], and HER2/neu-positive tumor [81]. Thun et al. also found a significantly lower incidence of colorectal and gastric cancer in patients treated with long-term aspirin, an NSAID that inhibits COX [82, 83]. The high COX-2 expression in cancer tissues and epidemiological data on the effect of COX inhibitors on carcinogenesis suggest that COX-2 inhibitors may have an effect on carcinogenesis. The role of COX-2 expression in carcinogenesis has also been studied in digestive cancer, including colorectal cancer. mPGES-1, an enzyme that converts PGH2 produced via COX-2 into PGE2, is strongly expressed in many tumor tissues, together with induced COX-2 expression [84]. Therefore, coupled action of COX-2 and mPGES-1 may increase PGE2 production in tumor tissues. The incidence of intestinal polyp is markedly reduced in Apc^Δ716^ mice with a defective EP2 gene, a PGE receptor. Therefore, signaling pathways involving PGE2 and other prostanoids downstream of COX-2 may play important roles in intestinal tumors [85].

Involvement of PGE2 in carcinogenesis occurs through several mechanisms. In tumor tissues of Apc^Δ716^ mice, COX-2 expression is significantly decreased, which indicates that PGE2 signaling via EP2 induces expression of COX-2. PGE2 produced downstream of COX-2 maintains overexpression of COX-2 by positive feedback [85]. Expression of an angiogenic factor, VEGF, and basic fibroblast growth factor (bFGF) is also induced via EP2; thus, induced COX-2 expression enhances PGE2 production and activates signals via EP2, resulting in enhanced angiogenesis [86, 87]. PGE2 signaling also induces activation of matrix metalloproteinases (MMPs), which release transforming growth factor-*α* (TGF-*α*), an epidermal growth factor receptor (EGFR) ligand, from cell membranes. TGF-*α* induces proliferative signals by binding to EGFR in tumor cells [88]. PGE2 stimulation also induces expression of amphiregulin, an EGFR ligand, and the protease ADAM17, which releases EGFR ligands from cell membranes [89]. PGE2 signals can also activate peroxisome PPAR*δ*.

Activation of Wnt, a secretory glycoprotein, induces expression of PPAR*δ*, and PGE2 signals induce PPAR*δ* transcriptional activity via activation of the PI3K/Akt pathway and inhibit apoptosis in tumor cells [90]. G proteins bound to ER2 receptors bind to axin with PGE2 stimulation, and formation of a complex between APC and *β*-catenin is inhibited. *β*-catenin is not phosphorylated, becomes stabilized, migrates to the nucleus, and induces transcription of Wnt-target genes. PGE2 also directly activates Wnt signals involved in carcinogenesis [91]. Thus, the COX-2/PGE2 pathway may be involved in carcinogenesis through various mechanisms. Prevention of carcinogenesis by COX-2 selective inhibitors has been attempted, but monotherapy is difficult because these inhibitors cause heart-related adverse reactions. For this reason, a combination of COX-2 inhibitors with current anticancer drugs may be the best approach to improve the therapeutic effect.

High expression of COX-2 is also found in cervical cancer, and COX-2 is found in cervical intraepithelial neoplasia (CIN), in addition to invasive cancer. Epidermal growth factor (EGF) activates mRNA and promoters of COX-2 in cancer tissues [92], and COX-2 induction by EGF is suppressed by inhibition of kinases including PI3K, mitogen-activated protein kinase (MAP2K), and p38 MAPK. COX-2 expression is also markedly increased in patients with lymph node metastases and invasion in the parametrium [93]. Many patients with cervical cancer, particularly adenocarcinoma, have COX-2 overexpression, and the effects of neoadjuvant chemotherapy were poorer and survival was shorter in patients with higher COX-2 levels [94]. Thus, COX-2 has an important role in carcinogenesis and COX-2 inhibitors may be effective as cancer therapy. In this context, clinical trials of tegafur-uracil (UFT) + cyclophosphamide + celecoxib are underway in patients with advanced recurrent cervical cancer.

COX-2 inhibitors also increase the sensitivity of cancer cells to drugs and radiotherapy, in addition to having a direct anticancer effect.* In vitro*, administration of anticancer taxanes was found to stabilize mRNA of COX-2 [95]. If this occurs* in vivo*, COX-2 may be implicated in acquisition of resistance to taxanes. In another* in vitro* study, COX-2 inhibitors were found to enhance the antitumor effects of cisplatin and paclitaxel [96], while COX-2 overexpression decreases radiosensitivity by inhibiting apoptosis induced by radiation, regardless of overexpression and induction of p53 protein prior to treatment [93]. These results suggest that COX-2 inhibitors may increase radiosensitivity, and this effect has been shown in tumor cells; however, COX-2 inhibitors also damaged normal proliferating cells, including the intestinal tract. Drugs such as celecoxib inhibit PG production by COX-2 in cancer tissues and specifically increase the sensitivity of tumor cells to radiotherapy [97, 98], but the efficacy is uncertain [99]. In general, these studies indicate that a combination of current therapy and COX-2 inhibitors may improve outcomes and result in fewer adverse reactions in treatment of cervical cancer. Clinical studies are required to examine these issues further.

## 7. Conclusion

New drug development is currently occurring slowly and discovery of new antitumor drugs may be facilitated by drug repositioning. In this process, it is important to analyze various pharmacological actions of existing drugs and to show a low incidence of a certain cancer epidemiologically in patients treated with these drugs. PPAR ligands, ritonavir, metformin, and COX-2 inhibitors may be effective for gynecologic tumors, but these drugs have yet to be used in clinical practice (Table 1). Use of these drugs in combination with current anticancer drugs may improve efficacy, reduce adverse reactions, and improve patient QOL. Further clinical and epidemiological studies are required to examine these issues.

## Figures and Tables

**Table 1:** Drug repositioning for treatment of gynecologic tumors.

Drug	Original target	New target
PPAR*γ* ligand	Type 2 diabetes, atherosclerosis	Ovarian cancer
Ritonavir	AIDS	Ovarian cancer
Metformin	Type 2 diabetes	Endometrial cancer
COX-2 inhibitor	Inflammation, pain	Cervical cancer
